# P12 Antibiotic prescribing in hospitalized patients with COVID-19 as a function of inflammatory markers in Wave 1 versus Wave 2: a systematic review

**DOI:** 10.1093/jacamr/dlac053.012

**Published:** 2022-05-31

**Authors:** Saif Abbas Chatoo, Emma Thomas-Jones, Philip Pallmann, Joanne Euden

**Affiliations:** 1 Cardiff University School of Medicine, Cardiff, UK; 2 Centre for Trials Research, Neuadd Meirionydd, Cardiff University, Cardiff, UK

## Abstract

**Background:**

Less than 10% of hospitalized cases in the United Kingdom during the first wave of the pandemic had bacterial coinfection, but approximately 75% were prescribed antibiotics contrary to NICE guidelines. We have evaluated the relationship between antibiotic prescribing and biomarker use, in hospitalized adult patients with COVID-19 in the UK, as synthesis defined by the pandemic timeline, particularly during ‘Wave 2’ is lacking. Clinical outcomes were compared in the context of antimicrobial stewardship. C-reactive protein (CRP), erythrocyte sedimentation rate (ESR), procalcitonin (PCT) and white cell count (WCC) were selected based on clinical relevance.

**Methods:**

Studies (*n* = 300) dated 2019–22 were identified via EMBASE and Web of Science databases, using relevant search terms. Wave 1 and Wave 2 parameters were defined by the Office for National Statistics. Literature selection was organized by the preferred reporting items for systematic reviews and meta-analyses (PRISMA). PROSPERO registration was commenced mitigating unplanned duplication. Diagnostics, prescribing, clinical outcomes and demographics were tabulated. Ten percent of studies were cross-checked.

**Results:**

Final selection criteria yielded 29 papers, of which only 4 were from Wave 2. Cohort and retrospective studies accounted for 69% and 80%, respectively. Heterogeneity of studies prevented a meta-analysis. Determining disease severity or coinfection was the focus of ( *n*= 6) studies. The papers referencing WCC (*n* = 12) established that leucocytosis, like elevated CRP, was an efficient marker for bacterial infections. Additionally, CRP >100 mg/L was associated with increased prescribing. Another common theme was cut-off values to escalate (*n* = 9) or de-escalate (*n* = 12) prescribing. In PCT papers (*n* = 14), common de-escalation cut-offs were 0.25 ng/mL and 0.50 ng/mL. Lower admission PCT was associated with decreased mortality, admission duration and ICU admission rate. ESR use was unevaluable as it was only mentioned in one case study. During Wave 2, the use of immunomodulatory therapy may have contributed towards lower inflammatory markers. Hospital stays decreased while ICU duration increased. In ICU patient studies (*n* = 4) biomarker testing was more frequent. The higher 0.50 ng/mL PCT cut-off was employed but with higher mortality and ventilation rate.

**Conclusions:**

More studies of Wave 2 cohorts are required. A weakness of the evidence base is that cohort study outcomes were reported in varying detail, a potential consequence of the need for rapidly published evidence. Nonetheless, PCT and CRP were demonstrated to be useful as prognosis indicators on hospital admission, and in timely antibiotic prescribing. Updated national guidelines should include standardized biomarker thresholds to improve antimicrobial stewardship.

